# Orange jasmine as a trap crop to control *Diaphorina citri*

**DOI:** 10.1038/s41598-019-38597-5

**Published:** 2019-02-14

**Authors:** Arthur F. Tomaseto, Rodrigo N. Marques, Alberto Fereres, Odimar Z. Zanardi, Haroldo X. L. Volpe, Berta Alquézar, Leandro Peña, Marcelo P. Miranda

**Affiliations:** 1 Department of Entomology, Fund for Citrus Protection (FUNDECITRUS), 14807-040 Araraquara, São Paulo Brazil; 2 Centre of Nature Sciences, Federal University of São Carlos (UFSCAR), Buri, São Paulo Brazil; 3 Departamento de Protección Vegetal, Instituto de Ciencias Agrarias (ICA/CSIC), C/Serrano, 115 dpdo, 28006 Madrid, Spain; 4 Instituto de Biología Molecular y Celular de Plantas (IBMCP), Consejo Superior de Investigaciones Científicas (CSIC), Universidad Politécnica de Valencia (UPV), 46022 Valencia, Spain

## Abstract

Novel, suitable and sustainable alternative control tactics that have the potential to reduce migration of *Diaphorina citri* into commercial citrus orchards are essential to improve management of huanglongbing (HLB). In this study, the effect of orange jasmine (*Murraya paniculata*) as a border trap crop on psyllid settlement and dispersal was assessed in citrus orchards. Furthermore, volatile emission profiles and relative attractiveness of both orange jasmine and sweet orange (*Citrus* × *aurantium* L., syn. *Citrus sinensis* (L.) Osbeck) nursery flushes to *D. citri* were investigated. In newly established citrus orchards, the trap crop reduced the capture of psyllids in yellow sticky traps and the number of psyllids that settled on citrus trees compared to fallow mowed grass fields by 40% and 83%, respectively. Psyllids were attracted and killed by thiamethoxam-treated orange jasmine suggesting that the trap crop could act as a ‘sink’ for *D. citri*. Additionally, the presence of the trap crop reduced HLB incidence by 43%. Olfactometer experiments showed that orange jasmine plays an attractive role on psyllid behavior and that this attractiveness may be associated with differences in the volatile profiles emitted by orange jasmine in comparison with sweet orange. Results indicated that insecticide-treated *M. paniculata* may act as a trap crop to attract and kill *D. citri* before they settled on the edges of citrus orchards, which significantly contributes to the reduction of HLB primary spread.

## Introduction

Asian citrus psyllid, *Diaphorina citri* Kuwayama (Hemiptera: Liviidae) is considered the main threat to orange production due to its ability to transmit the putative causal agents (‘*Candidatus* Liberibacter spp.’) of huanglongbing (HLB), the most destructive citrus disease worldwide^1^. There is yet no effective cure for HLB, which affects all commercial citrus varieties and produces billion dollar losses to citriculture^2^. Current management of HLB is based on the prevention of citrus tree infection through planting of healthy nursery trees, inspections and eradication of symptomatic trees and control of its vector *D. citri* using insecticides^3^.

HLB spread is mainly associated with constant *D. citri* short and long-range flight movements by psyllid adults^4–7^. Primary HLB infection occurs mainly on orchard borders^8,9^ where the psyllid prefers to settle^10^. Despite the intensification of chemical control of *D. citri* on grove borders in recent years, concomitant reductions of HLB infections is limited because of the intense and constant influx of migrating psyllids^9,11^. Considering the HLB ‘edge effect’ and the difficulties to avoid primary infections, it is important to consider the development of novel and suitable alternative tactics to be incorporated into HLB management programs.

The trap cropping tactic has been studied for the integrated pest management of many insect pests, including aphids, leafhoppers, planthoppers, and whiteflies, which are important hemipteran vectors of plant diseases^12^. Trap crops are plants used to attract insects or other organisms in order to protect important economic crops from direct damage or indirect damage related to vector-transmitted diseases^13^. The trap plant could act as a barrier, protecting the crop of interest from the pest or vector, by concentrating them on attractive leaves or organs where their control could be more efficient and specifically applied. Shelton and Badenes-Perez^12^ proposed a broader definition of trap crops, by including plants that are, per se or via manipulation, responsible for attracting, diverting, intercepting, and/or retaining pests or vectors, and consequently, decreasing the damage inferred to the main crop. Moreover, in order to increase the efficiency of controlling pests, trap crops can be associated with insecticides. Successful trap cropping strategies to manage pests have been described previously. For example, using alfalfa (*Medicago sativa* L.) as a trap crop reduced populations of the Western tarnished plant bug *Lygus hesperus* Knight (Hemiptera: Miridae) in cotton (*Gossypium hirsutum* L.)^14,15^. More recently, borders of transgenic Rainbow papaya (*Carica papaya* L.) plants resistant to the *Papaya ringspot virus* were used as trap crop to reduce the spread of viruliferous aphid vectors to non-transgenic papaya plants in Hawaii^16^.

Preliminary field studies in São Paulo (SP) State, Brazil, showed that the use of suitable host plants for *D. citri* as barriers reduced the number of marked psyllids recaptured on yellow stick traps deployed on citrus trees^7^. The lowest recapture rates were recorded when orange jasmine [*Murraya paniculata* (L.) Jack, syn. *Murraya exotica* L.] was used as a barrier, envisaging its potential use as a trap crop to manage HLB. Orange jasmine is a preferred host for *D. citri* in comparison with other rutaceous host plants^17,18^ and this effect has been related to its volatiles^19^. Although orange jasmine can be infected with ‘*Candidatus* Liberibacter asiaticus’ (CLas), bacterial titer is much lower in this host than in *Citrus* species and cultivars and decreases over time, probably due to the lower multiplication rates in the former, being CLas infections usually transient in orange jasmine^20,21^. Moreover, recent results showed that *D. citri* could not acquire CLas upon feeding and developing on CLas-qPCR positive orange jasmine seedlings^22^. These results indicate that orange jasmine is a poor host for CLas (as well as a ‘dead-end’ host plant^23^) and suggest that it may be used as a potential trap crop, attracting psyllids which could be controlled in these plants by insecticides, thus limiting spread of HLB. In this study, the effect of orange jasmine as a border trap crop on *D. citri* settlement and dispersal was assessed in sweet orange orchards. Additionally, the volatile emission profiles and relative attractiveness of both orange jasmine and sweet orange flushes to *D. citri* were investigated. The results of this study may help citrus growers to control immigrating psyllids that arrive to the edges of citrus orchards from external inoculum sources and thus limit HLB incidence inside citrus orchards.

## Results

### Effects of orange jasmine as a trap crop on *Diaphorina citri* natural infestation and HLB incidence

**Figure 1:**
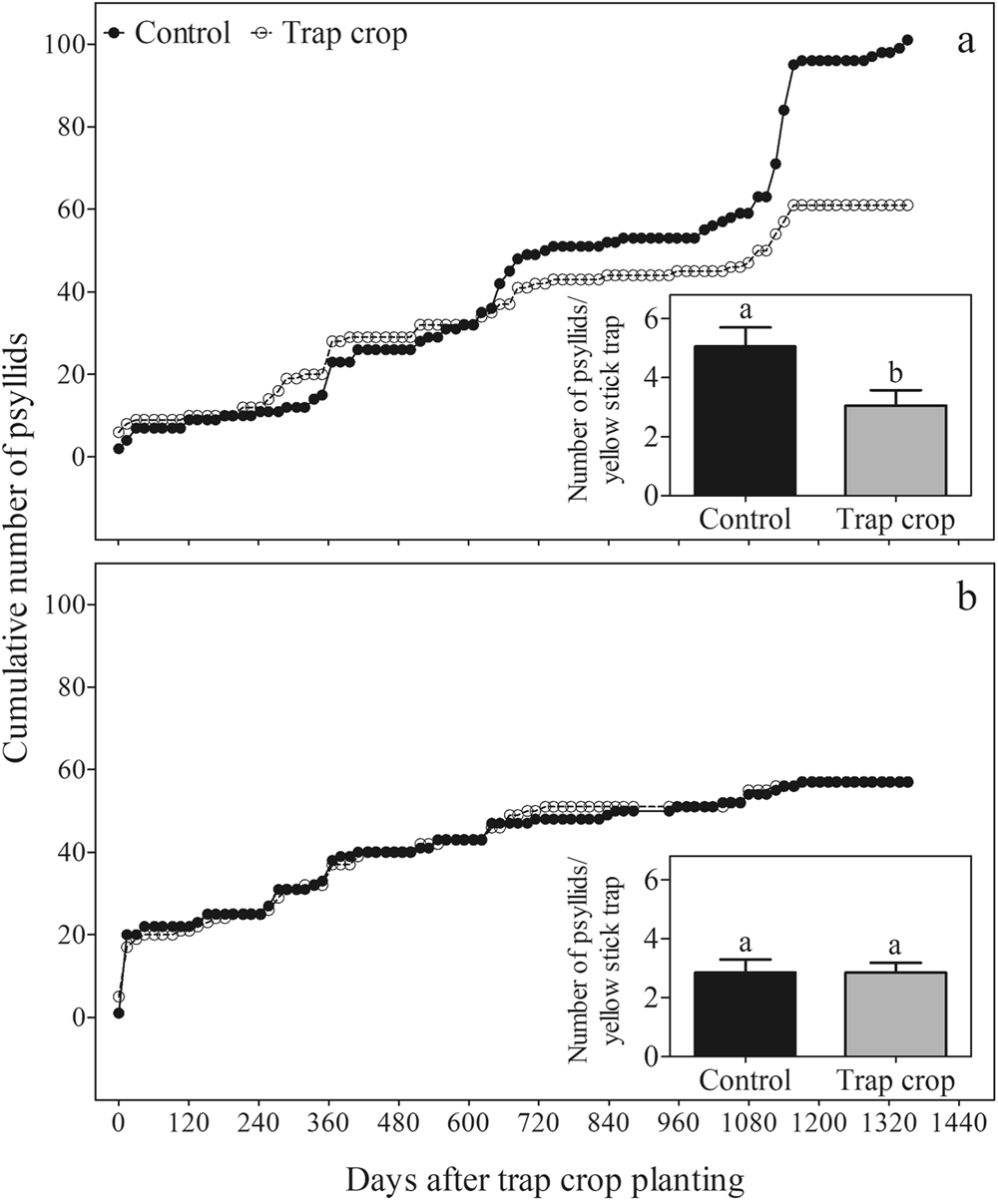
(Miranda) Cumulative number of *Diaphorina citri* collected on yellow stick traps placed in citrus trees located in plots with or without (control) orange jasmine trap crop on the border over a 45-month survey. Data from two experimental areas, (**a**) area A (new planting, 6 months old) and (**b**) area B (established orchard, 7 years old). Bar graphics represent the cumulative mean number of *D. citri* (±standard error) after 45 months of survey. Means followed by the same letter in the small bar graphics do not differ statistically (*P* < 0.05).

The presence of orange jasmine as a trap crop on the edge of a new (6-month-old) citrus orchard (Area A, Fig. 1a) reduced incidence of *D. citri* in the orchard, when compared to the control, over a 45-month survey (d.f. = 1; *P* = 0.0030). In this area significantly fewer psyllids were captured on yellow sticky traps placed in citrus trees bordered on the east side by the trap crop compared to identical traps placed in control trees bordered on the east side by fallow mowed grass (F = 5.74; d.f. = 1, 38; *P* = 0.0216) (Fig. 1a). In contrast, when the orange jasmine trap crop was planted on the edge of well established (7-year-old) citrus plots (Area B, Fig. 1b), cumulative trends in numbers of psyllids captured on yellow sticky traps placed in trees within the plots did not differ significantly, regardless of the presence or absence of an orange jasmine trap crop on the orchard edge (d.f. = 1; *P* = 0.8196), and the total numbers of psyllids recorded on the yellow sticky traps in both the trap crop and control treatments over 45 months did not differ significantly (F = 0.00; d.f. = 1, 38; *P* = 1.0000) (Fig. 1b).

The frequency of *D. citri* detection also varied between new and established citrus orchards. In new citrus plots (area A), the frequency of psyllid detection in the control treatment (40.6%) was significantly higher than in the trap-crop treatment (25.7%) (χ^2^ = 4.377; d.f. = 1; *P* = 0.0364). In contrast, in the 7-year-old citrus plots (area B), the frequency of *D. citri* detection in each treatment was similar (control: 25.3%; trap crop: 27.3%; χ^2^ = 0.026; d.f. = 1; *P* = 0.8717).

Regarding the assessments of ‘*Ca*. L. asiaticus’ or ‘*Ca*. L. americanus’-positive trees in the new citrus orchard, the HLB incidence in the control plot was ~2.8-fold (control: 1.4%; trap crop: 0.5%) and ~1.8-fold (control: 2.8%; trap crop: 1.6%) higher than on orange jasmine trap crop treatment, for assessments performed on May 2014 and on January 2015, respectively.

### Effects of orange jasmine as a trap crop on *Diaphorina citri* settlement and dispersal

**Figure 2:**
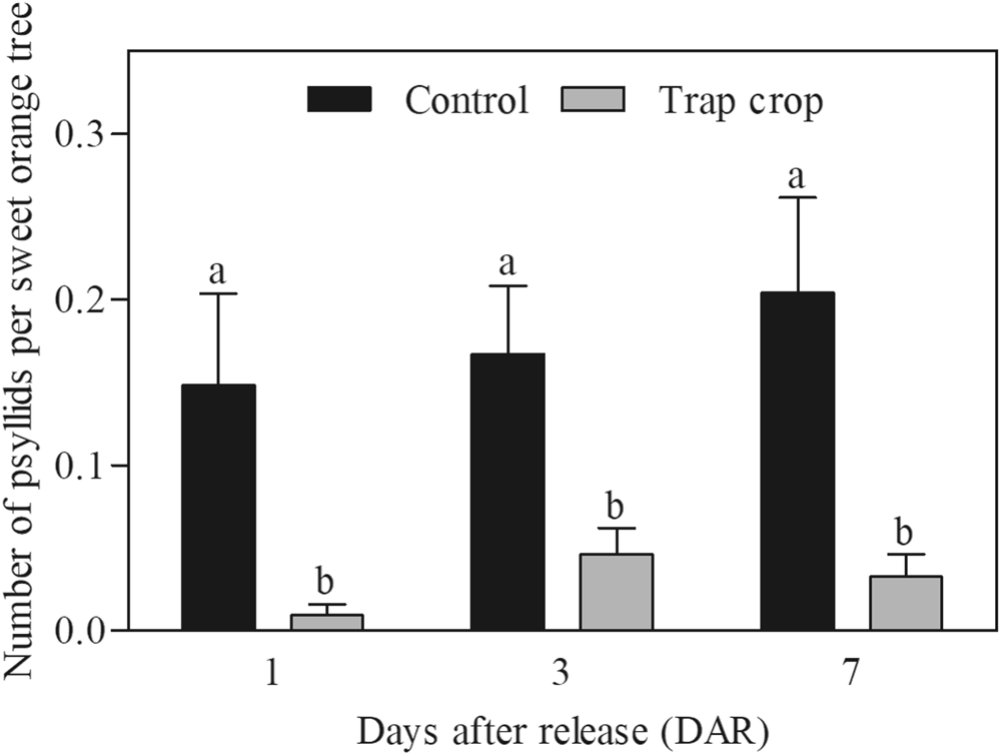
(Miranda) Number of marked *Diaphorina citri* (mean ± standard error) found on ‘Pera’ sweet orange trees located on plots with orange jasmine trap crop or fallow field (control) over time (DAR). Means followed by the same letter at each time did not differ statistically (*P* < 0.05).

To further investigate orange jasmine trap crop effect on *D. citri* settlement and dispersal, adult psyllids were marked with different colors of fluorescent powder and released near the experimental sweet orange orchard with and without trap crop plots. Trap crop plots showed a significant reduction in the number of psyllids that settled on citrus trees compared to control plots at 1 (F = 12.28; d.f. = 1, 430; *P* = 0.0005), 3 (F = 9.13, d.f. = 1, 430; *P* = 0.0027) and 7 (F = 15.42; d.f. = 1, 430; *P* = 0.0001) days after release (Fig. 2). Overall, the trap crop treatment provided a significant reduction in the number of *D. citri* adults that settled on citrus trees compared to the control (χ^2^ = 4.13; d.f. = 1; *P* = 0.0421), independently of time (χ^2^ = 2.86; d.f. = 1; *P* = 0.0908) and treatment and time interaction (χ^2^ = 0.17; d.f. = 1; *P* = 0.6768).

**Figure 3:**
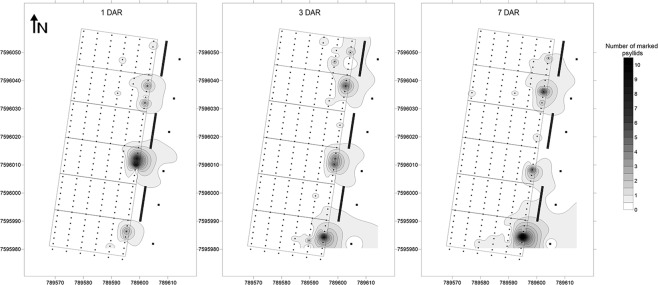
(Miranda) *Diaphorina citri* infestation maps at 1, 3 and 7 days after release (DAR). Data were obtained with the sum of psyllids found on citrus trees in the first, second and third replicate of the experiment to assess the effect of orange jasmine as a trap crop on *D. citri* settlement and dispersal. Each black dot represents a citrus tree. Black squares at the right side of map are the insect release platforms. The three groups of thick black lines represent the orange jasmine trap crop. Numbers at the bottom and left side of map are the Universal Transverse Mercator (UTM) coordinates from 22 k zone.

Assessments on release platforms showed that approximately 90% of the released psyllids had taken off from the platforms at 1 day after release (DAR) and no psyllids were found there by 3 DAR. In general, 83% of all marked psyllids that were found in the experimental area were in the first citrus row regardless of the assessment time (Fig. 3). Overall, 150, 205 and 56 marked psyllids (out of 16800) were found in the experimental area at 1, 3 and 7 DAR, respectively. At all inspection times, most psyllids (94.3, 78.7 and 86.3% at 1, 3 and 7 DAR, respectively) were found on control plots. Psyllids released in front of orange jasmine plots were mostly restricted to the first citrus row. In contrast, individuals released in front of control plots were found mainly in the first four citrus rows, and one psyllid reached the last citrus row (35 m from the release platforms).

**Figure 4:**
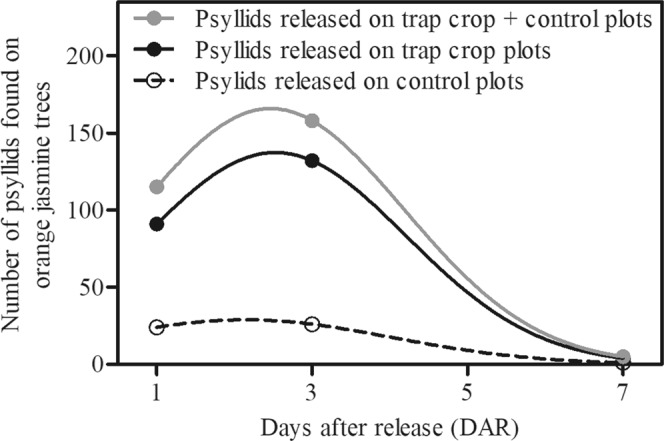
(Miranda) Number of marked psyllids found on orange jasmine trees according to the plots in which the insects were released: trap crop + control, trap crop and control. Visual assessments were performed at 1, 3 and 7 days after release (DAR). Observed data were fitted to Gaussian models (lines) with the equations y = 165.90 × exp(−0.50 × ((x-2.47)/1.71)^2^), y = 137.3 × exp(−0.50 × ((x-2.53)/1.68)^2^), and y = 28.88 × exp(−0.50 × ((x-2.14)/1.87)^2^) for psyllids released on trap crop + control, trap crop and control plots, respectively.

Visual assessments on orange jasmine trees at 1 and 3 DAR indicated the presence of 115 and 158 marked *D. citri*, respectively (Fig. 4). Few psyllids (5 individuals) were found on orange jasmine trees at 7 DAR. In addition, among all psyllids that were found on orange jasmine trees, 19% were individuals released in the control treatments.

### Insecticide effect on *Diaphorina citri* mortality

**Figure 5:**
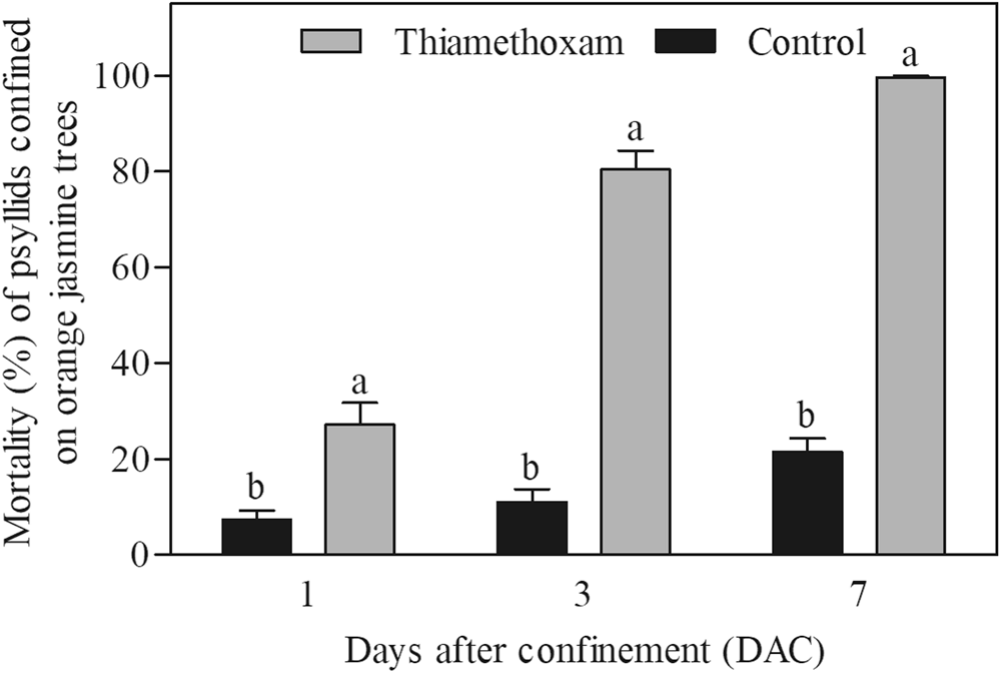
(Miranda) Mortality (%) (mean ± standard error) of *Diaphorina citri* adults confined on orange jasmine trees previously treated with systemic insecticide (thiamethoxam) or untreated (control) over time after psyllid confinement (DAC). Means followed by the same letter at each time do not differ statistically (*P* < 0.05).

Psyllid mortality was significantly higher for psyllids confined on orange jasmine trees treated with thiamethoxam compared to the untreated control (log rank: 322.00; d.f. = 1; *P* < 0.0001), considering the whole assessment period. The same results were observed when comparing the mortality rates at 1 (F = 24.79; d.f. = 1, 57; *P* < 0.0001), 3 (135.67; d.f. = 1, 57; *P* < 0.0001), and 7 (F = 209.14; d.f. = 1, 57; *P* < 0.0001) days after confinement (DAC) (Fig. 5).

### Olfactometric assays and volatile emission analysis

**Figure 6:**
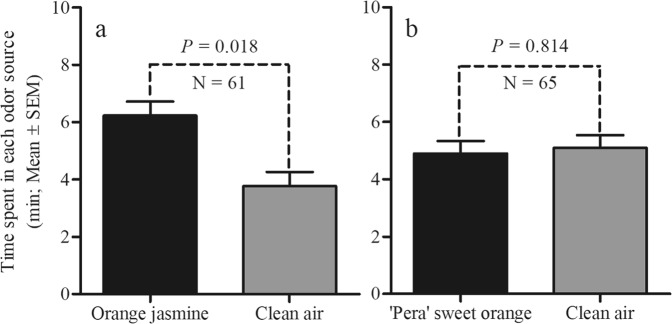
(Miranda) Responses of *Diaphorina citri* females, tested in a 4-arm olfactometer, to volatiles of two sets of odor sources [orange jasmine × control (clean air)] (**a**) and [‘Pera’ sweet orange × control (clean air)] (**b**). Bars represent the mean time spent by *D. citri* in each odor source (±standard error).

**Figure 7:**
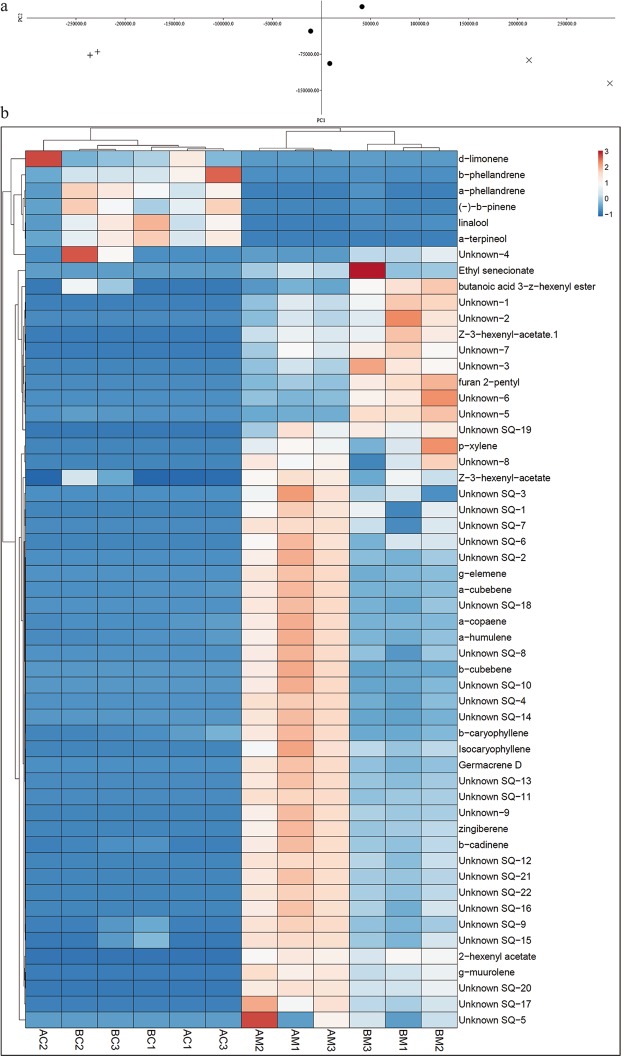
(Miranda) (**a**) Representative Principal Component Analysis (PCA) of MetAlign output scan peak values resulting from GC-MS of orange jasmine and sweet orange flushes. + corresponds to orange jasmine, X to orange and dots (•) to quality control samples (mixtures of flushes from both genotypes). PC1 explained at least 75% of the variance at two independent replicate dates. (**b**) Heatmap, generated with ClustVis, showing the corrected areas of selected volatile compounds emitted by orange jasmine (AM1, AM2, AM3, BM1, BM2, BM3) and sweet orange (AC1, AC2, AC3, BC1, BC2, BC3) flushes. Representation corresponds to three biological replicates (named 1, 2, 3) for each genotype (M, orange jasmine; C, orange) at two different sampling dates (A and B).

In 4-arm olfactometer assays, results indicated that *D. citri* females spent more time on orange jasmine than on the clean air fields (V = 1270.00; d.f. = 1, 60; *P* = 0.0185) (Fig. 6a). However, no significant effect was observed on the *D. citri* female foraging activity to the ‘Pera’ sweet orange vs. clean air (V = 1036.00; d.f. = 1, 64; *P* = 0.8139) (Fig. 6b). This different *D. citri* behavior to sweet orange and orange jasmine suggests different volatile profiles between both genotypes. To gain insight into the attractiveness of orange jasmine volatiles, comparative untargeted volatile analysis of sweet orange and orange jasmine flushes was performed. Principal component analysis revealed two separated clustering groups, one for each studied genotype (Fig. 7a). PC1 explained at least 75% of the variance at two independent replicate dates. Area integration of peaks corresponding to relevant loadings at both sampling dates (detailed in Supplementary Table 1) revealed important quantitative and qualitative differences in volatiles emitted from both genotypes (Fig. 7b). For example, 37 compounds (of which 26 are sesquiterpenes) were detected only in the orange jasmine emission profile. On the other hand, the emission of monoterpenes characteristic of sweet orange leaves, such as α- and β-phellandrene, d-limonene and linalool, was highly reduced in orange jasmine.

## Discussion

In the current study, we evaluated first the use of orange jasmine as a trap crop in two types of orange orchards, recently planted and several years old, that differed in plant height and biomass (new orchard: 1 m-tall; well established orchard: 3 m-tall; Supplementary Fig. 1). The presence of orange jasmine as a trap crop in the border of new citrus orchards caused overall reductions of 40% in the accumulated number of psyllids captured (Fig. 1a) and 83% in the number of psyllids settled on citrus trees (Fig. 2), when compared to the fallow field plots (control). Moreover, in the control plot the frequency of *D. citri* detection was 1.6-fold higher than in the orange jasmine plot (Fig. 1a). Similar insect reduction rates associated to the trap crop use were obtained after planting green leaf desmodium [*Desmodium intortum* (Mill.) Urb.] and *Brachiaria* cv. Mulato II as ‘push’ and ‘pull’ crops, respectively, on maize (*Zea mays* L.), in order to reduce the fall armyworm [*Spodoptera frugiperda* (J.E. Smith)] (Lepidoptera: Noctuidae) infestation and damage^24^. Likewise, Stern *et al*.^14^ demonstrated a reduction in the *L. hesperus* movement into cotton in California (USA), when alfalfa was intercropped with cotton.

Regarding *D. citri* spatial distribution, most psyllids were found in the first rows of citrus (Fig. 3) regardless of the treatment and assessment period, reinforcing psyllid preference for trees located in the border of the orchards^10,25,26^. Psyllids released in front of the trap crop plots did not disperse further than the first row of citrus, whereas individuals released in front of control plots were able to cross the first row and were present in almost all rows. On the other hand, a high number of individuals were observed on orange jasmine trap crop during the first assessments (Fig. 4), including insects that were released on control plots. These results may be explained by a strong attraction effect associated to the orange jasmine trees, which could have reduced psyllid movement into the orchard, and suggest that the psyllid has the ability to detect and move to the preferred host rather than performing a passive random dispersal. This hypothesis is in accordance with previous studies that report the importance of visual and olfactory cues in the host plant finding ability of *D. citri*^27–29^.

The low effect of orange jasmine trap crop on *D. citri* infestation when tested on well-established citrus orchards also supports the hypothesis of visual and olfactory cues for *D. citri* behavior. Probably, large differences in plant size between orange jasmine and citrus trees on mature plots may lead to higher presence of visual/olfactory cues from citrus trees than from orange jasmine plants, thus explaining the absence of trap crop effect over the *D. citri* population in this case. According to Shelton and Badenes-Perez^12^, a successful trap crop relies on the combination of trap crop (e.g. size, phenology, and attractiveness) and pest characteristics.

To evaluate the effectiveness of orange jasmine olfactory cues as *D. citri* attractants, 4-arm olfactometric assays were performed (Fig. 6). An increase of ~30% in *D. citri* preference to orange jasmine volatiles in relation to that of ‘Pera’ sweet orange was observed. High attractiveness of orange jasmine flushes to *D. citri* has been also observed in Y-olfactometer^19^. Therefore, our olfactometric data reinforces the *D. citri* preference to volatiles from orange jasmine instead of sweet orange flushes. In this sense, the orange jasmine odors plume could contribute for psyllid mobility towards orange jasmine, which increases the *D. citri* settling preference to this host, as observed in field experiments.

Regarding the volatile analysis, we found that orange jasmine volatile profile is clearly distinguishable to that of sweet orange (Fig. 7). Then, analyses were conducted to identify which compounds were relevant to make this distinction either quantitatively or qualitatively (Supplementary Table 1). Green leaf volatiles, such as hexenyl acetates^30^, were more emitted by orange jasmine than by sweet orange leaves, as previously reported^31^. Monoterpene emission was also different between genotypes, being all them less abundantly emitted by orange jasmine. Lower emission of d-limonene and β-ocimene was also found in orange jasmine in relation to lemon (*Citrus* × *limon* (L.) Osbeck), rough lemon (*Citrus* × *taitensis* Risso syn. *Citrus jambhiri* Lushington), sweet orange, grapefruit (*Citrus* × *aurantium* L. syn. *Citrus paradisi* Macfad.) and *Citrus* × *macrophylla* Wester in other studies^19,31,32^. Orange jasmine emits less β-pinene and linalool than ‘Red Rio’ grapefruit, ‘Meyer’ lemon (*Citrus* × *limon*) and ‘Valencia’ sweet orange^19,31^. Most of the relevant compounds emitted by orange jasmine corresponded to sesquiterpenes, usually absent or poorly emitted by *Citrus* leaves^19,31–33^. Nearly all of the identified sesquiterpenes have been reported before as emitted by orange jasmine leaves^19,31,32^. These results are in accordance with the highest attractiveness of psyllids to orange jasmine volatiles in the olfactometer device (chemical cues) and field experiments (chemical and visual cues). Delving in this area until determination of which compound/s are responsible for higher attraction of orange jasmine leaves to *D. citri* would allow the development of more efficient traps to control/monitor insect populations.

Besides reducing *D. citri* population and its spread, the presence of orange jasmine trap crop at the edge resulted in 43% reduction in HLB incidence at the orange orchard. Similarly, a cereal border crop reduced in 51.5% the incidence of Bean yellow mosaic virus (transmitted by aphids) compared to fallow fields in narrow-leaved lupin (*Lupinus angustifolius* L.)^34^. Despite orange jasmine has been reported as a CLas host, bacterial titers are much lower in this host than on citrus trees^20,21,35–37^, and consequently, it may be considered as an irrelevant inoculum source epidemiologically. Additionally, it is known that *D. citri* adults are less efficient than nymphs to acquire CLas^38–40^, and the transmission process demands a latent period of at least 7–10 days before psyllids become bacteriliferous^41^. Moreover, at 7 DAR the number of psyllids on orange jasmine plants decreased 97% (Fig. 4), probably due to the treatment with thiamethoxam. This observation was confirmed with data from the experiment in which psyllids confined on thiamethoxam-treated orange jasmine plants presented mortality close to 100% at 7 DAC (Fig. 5). The low number of released psyllids recaptured on citrus trees (0.3%) in comparison to that of insects recaptured when no trap crop was used^7,25^ supports that thiamethoxam treated-orange jasmine acted as a ‘sink’ for *D. citri*, attracting, killing and consequently, reducing psyllid movement into the citrus orchard. Therefore, it is reasonable to consider that the acquisition of CLas from a psyllid that landed on treated orange jasmine and subsequent inoculation in heathy citrus trees from a commercial orchard would be close to zero.

In summary, this study demonstrated for the first time that *M. paniculata* treated with insecticides may act as a trap crop to attract and kill *D. citri* before settling on the border of citrus orchards. However, the use of orange jasmine as a trap crop should be implemented before, at the same time or soon after citrus tree planting. Moreover, our work opens the possibility of performing studies assessing the trap crop integration with other tactics (e.g. kaolin), as a ‘push’ and ‘pull’ strategy, which could decrease further infestation rates inside citrus orchards. Finally, in order to avoid the use of insecticides, a genetically modified trap crop, able to interfere with *D. citri* survival, could be used in the citrus edges to attract and kill *D. citri*. An analogous approach has been used in Hawaii, where borders of transgenic papaya plants resistant to *Papaya ringspot virus* reduced the spread of aphid-vectors and then viral incidence on non-transgenic papaya crops^16^.

## Methods

### Effects of orange jasmine as a trap crop on *Diaphorina citri* natural infestation and HLB incidence

The study was carried out from October 2011 to July 2015 in two areas from a commercial orchard located in Matão, SP State, Brazil subjected to HLB control. The area A (21.60806°S, 48.42611°W) was located in a plot of ‘Hamlin’ sweet orange (*Citrus* × *aurantium* L., syn. *Citrus sinensis* (L.) Osbeck) trees (6 months old; ~1.0 m-tall; Supplementary Fig. 1a,b) grafted on ‘Swingle’ citrumelo [*Citrus* × *aurantium*, syn. *Citrus paradisi* Macfad. × *Citrus trifoliata* L., syn. *Poncirus trifoliata* (L.) Raf.] with spacing of 7.5 × 2.5 m. The area B (21.58556°S, 48.54556°W) was located in a plot of ‘Valencia’ sweet orange trees grafted on Rangpur lime ‘*Citrus limonia*’ Osbeck (7 years old; ~3.0 m-tall; Supplementary Fig. 1c,d) with spacing of 6.5 × 2.8 m. Both areas were historically subjected to continuous influx of *D. citri* from neighbor areas with high HLB incidence.

Each area was divided into two plots of 100 × 120 m. In one plot of each area, the orange jasmine trap crop was planted on east (area A) and north (area B) edges, 20 m from the first citrus tree, in two double-rows (1 m separated) with spacing of 0.4 × 0.4 m per plant. In total, 325 orange jasmine trees (~0.6 m-tall) were planted per row forming a canopy with 2 m in width and 120 m in length. The remaining plots of each area were maintained as fallow mowed grass and used as controls.

Orange jasmine trees were treated with drench applications of thiamethoxam (Actara^®^ 250 WG, Syngenta Proteção de Cultivos Ltda., Paulínia, SP, Brazil) 10 days before planting (0.25 g tree^−1^) and every 70 days thereafter (0.31 g of active ingredient per meter of tree height). In addition, foliar applications of insecticides with different modes of action (pyrethroid, organophosphate and neonicotinoid) were applied at interval of 14 days to both, trap crop and citrus orchards. The trap crop was fertilized every 60 days with NPK (10-10-10) at 100 g tree^−1^.

Psyllid populations were monitored by placing yellow sticky traps (30 × 10 cm) (ISCA^®^ Technologies, Ijuí, RS, Brazil) in 20 sweet orange trees located at 25 and 30 m from the trap crop. The same trap arrangement was used for the fallow field plots. Traps were assessed and replaced fortnightly. The number of *D. citri* adults was recorded on each trap.

Two visual assessments (May 2014 and January 2015) of HLB-symptomatic trees were performed in all citrus trees from each new citrus plot (area A) and leaf samples from suspected HLB-infected trees were tested by quantitative polymerase chain reaction (qPCR) for the presence of CLas or ‘*Ca*. L. americanus’^42^.

### Effects of orange jasmine as a trap crop on *Diaphorina citri* settlement and dispersal

**Figure 8:**
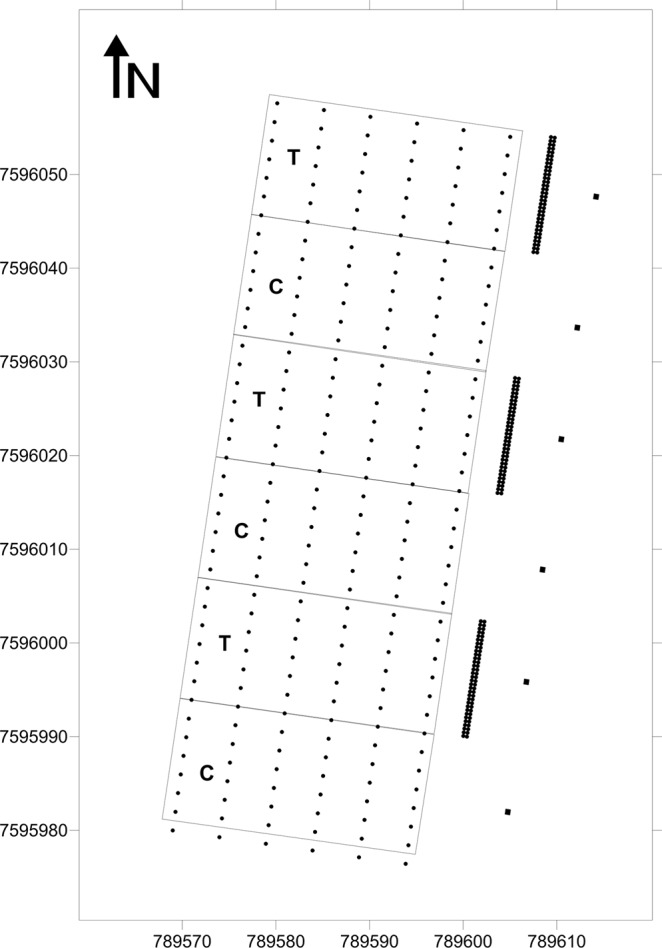
(Miranda) Schematic view of the experimental area used to evaluate the effect of orange jasmine as a trap crop on *Diaphorina citri* settlement and dispersal. Each black dot represents a citrus tree; and black squares at the right side of map are the insect release platforms. The three groups of thick black lines represent the orange jasmine trap crop. Letters “T” and “C” in each plot represent the trap crop and control treatments, respectively. Numbers at the bottom and left side of map are the Universal Transverse Mercator (UTM) coordinates from 22 k zone.

The experiment was carried out in an experimental area of ‘Pera’ sweet orange (2 years old and ~1.5 m-tall) grafted on Rangpur lime with spacing 5 × 2 m, located in Araraquara, SP, Brazil (21.71500°S, 48.20083°W). The area was divided into three blocks and each one split into two plots (36 trees plot^−1^) of 30 × 13 m (Fig. 8). Treatments were defined according to the presence or absence of orange jasmine as a trap crop in the citrus orchard border. In each plot, 65 nursery *M. paniculata* trees (1.5 m-tall) were planted, 4 m from the first citrus tree, in a double-row spacing of 0.4 × 0.4 m, forming a canopy with 0.4 m in width and 13 m in length (Fig. 8). Control plots were maintained as fallow mowed grass. The orange jasmine trees were treated with drench application of thiamethoxam (0.47 g tree^−1^) 10 days before planting. In order to ensure the insecticide efficacy, 10 nursery *M. paniculata* trees treated with thiamethoxam and 10 untreated were planted on the central west side of the experimental area at 2 m from the last citrus row. In these plants, groups of 10 adult psyllids (10–15 days old) were confined on a young shoot of each orange jasmine tree using sleeve cages, and *D. citri* mortality was assessed at 1, 3 and 7 days after the beginning of the experiment. Psyllid confinements on untreated orange jasmine trees were used as a control.

Adult psyllids, obtained from a colony free of ‘*Ca*. Liberibacter spp.’, maintained for several generations on orange jasmine seedlings at Fundecitrus (Araraquara, SP State, Brazil), were marked with different colors of fluorescent powder (Day-Glo Color Corp., Cleveland, OH, USA)^43^ in order to differentiate psyllids released on orange jasmine and fallow plots. Before release, marked insects were acclimated for 48 h on orange jasmine seedlings. Field release was conducted as described by Tomaseto *et al*.^7^ in 1.5 m-tall platforms located on the east side of the area at 10 m from the first citrus trees of each plot.

The experiment was replicated three times, and psyllid releases were always performed in the afternoon (~15:00), which is the period of the highest *D. citri* flight activity^27,44,45^, with 800–1000 marked psyllids per plot. The number of settled psyllids was assessed by visual inspection on 24 citrus trees (central trees of each row) from each plot (Fig. 8), at 1, 3 and 7 days after release (DAR).

Temperature and rainfall were monitored using a weather station (Vantage Pro2 6152; Davis Instruments, Hayward, CA, USA) 20 m far from the experimental area. For the first replicate, the mean temperature ranged from 22.0 to 25.1 °C with total precipitation of 19.2 mm, while for the second and third replicates, temperature and rainfall values ranged from 23.3 to 27.8 °C with 64.5 mm and from 21.7 to 24.06 °C with 6.1 mm, respectively (Supplementary Fig. 2).

### Olfactometric assays

The assays were carried out in a climate-controlled room at temperature 25 ± 2 °C, relative humidity 65 ± 10%, and 3000 lux luminosity. The preference of *D. citri* toward volatiles was investigated using a 4-arm olfactometer (30.0 × 30.0 × 2.5 cm in length, width, and height, respectively), adapted with a yellow acrylic floor arena, essentially as described in Zanardi *et al*.^46^. Individual constant (0.1 L min^−1^) charcoal-filtered humidified airflow, provided by an oil-free air compressor (Schulz MSV6, Schulz, Joinville, SC, Brazil) converged through 0.635 cm-diameter individual PTFE tubes (Sigma-Aldrich, Bellefonte, PA, USA) to the center of the acrylic arena. A single mated 7–15-day old female was released on the center of the arena. Psyllids that did not perform a choice after 5 min were recorded as “no response” in the analysis. In case of response, 10 min were allowed to observe the time spent in each one of the four odor fields. For each psyllid (replicate), the time spent in each odor source was recorded. Data were collected from 10:00 a.m. to 4:00 p.m. from five different assay days and a total of 61 (orange jasmine × clean air) and 65 (‘Pera’ sweet orange × clean air) replicates were used. Two of the four possible arms received plant volatiles whereas the two remaining arms received clean air^33^. Odor sources were switched each assay, and the arena was rotated after each responding insect to prevent bias. Orange jasmine and ‘Pera’ sweet orange nursery trees (~1 year old) to be used as odor sources were pruned 20 days before assays to stimulate the emergence of new shoots in a greenhouse.

### Volatile emission analysis

New flushes from 1m-tall *M. paniculata* and *Citrus* × *aurantium* sweet orange trees were detached and kept 30 min with the petioles submerged in water to acclimate. Subsequently they were enclosed in 20 mL screw-cap Pyrex tubes carrying a Teflon septum on the top and containing 1 mL of milli-Q water (for avoiding leaf hydric stress) and maintained 24 h at a controlled temperature of 22 °C. Volatile capture and GC-MS analysis were performed using a 6890 N gas chromatograph (Agilent Technologies Inc., Las Rozas, Spain) coupled to a Thermo DSQ mass spectrometer equipped with a DB-5 ms (Agilent J & W Columns) column (60 m × 0.25 mm i.d. × 1.00 μm film) as described before^33^. In short, SPME fiber (100 μm poly (dimethyl) siloxane/divinylbenzene (Supelco Inc., Bellefonte, PA) was exposed for 30 min at 22 °C and immediately afterwards transferred to GC injector (220 °C) where thermal desorption was prolonged to 4 min. The GC interface and MS source temperatures were 260 °C and 230 °C, respectively. Oven programming conditions were 40 °C for 2 min, 5 °C min^−1^ ramp until 250 °C, and a final hold at 250 °C for 5 min. Helium was the carrier gas at 1.5 mL min^−1^ in the splitless mode. Data was recorded in a 5975B mass spectrometer (Agilent Technologies Inc., Las Rozas, Spain) in the 35‒250 m/z range at 7 scans, with electronic impact ionization at 70 eV.

Samples from orange jasmine and sweet orange were analyzed by triplicate and at two different sampling dates. At each sampling date, a quality control sample (composed by mixtures of both genotypes) was analyzed by triplicate. Datasets were processed independently by the MetAlign software (www.metalign.nl) for full mass spectral alignment, baseline correction, noise estimation, and ion-wise mass spectral alignment. The MetAlign output scan peak values were corrected by leaf fresh weight and volatile accumulation time and then subjected to PCA analysis with Past3.17 software (folk.uio.no/ohammer/past). Significant scans were manually analyzed on TIC chromatograms to quantify the area and tentatively identify compounds by matching the acquired mass spectra with those stored in reference libraries (NIST, MAINLIB, REPLIB). The area for each compound was corrected by leaf fresh weight and volatile accumulation time and used to generate a heat-map using ClustVis^47^.

### Data analysis

Data from the commercial and the experimental citrus orchards were analyzed by Poisson generalized linear mixed models (GLMM) using the “*glmmADM*”^48^ (zero-inflated) and the “*lme4*”^49^ packages, respectively. Treatment was considered as fixed effect, while each assessment dates (commercial citrus orchards) or repeated measures on assessed trees (experimental orchard) as random. Time was considered fixed effect on experimental orchard data. The effect of treatment, time and interaction was assessed by likelihood-ratio tests (*P* < 0.05) between a full and a reduced model. In order to compare the effect of trap crop on cumulative mean number (commercial citrus orchards) or on counts of marked psyllids on citrus trees on each independent assessment time (experimental orchard), data were analyzed by a quasi-Poisson generalized linear model (GLM)^50^. Goodness-of-fit was assessed through half-normal plots with simulation envelopes using the “*hnp*” package^51^. In case of significant differences, means were separated by computing the 95% confidence intervals for linear predictors using the “*lsmeans*” package^52^. A 2 × 2 chi-squared contingency table was used to determine the treatment effects on frequency of *D. citri* detection (percentage of assessments with psyllid detection in relation to total number of assessments) in each commercial orchard plot. In order to analyze the efficacy of systemic insecticide applied on orange jasmine trees, survival rates in the whole assessment time were compared by a log-rank test (*P* < 0.05) and survival data at each time after release were compared by a quasi-binomial GLM. Infestation maps were generated using the Surfer^®^ software (Golden Software Inc., Golden, CO, USA) by the inverse of square of the distance interpolation method, considering the sum of insects found in all replicates.

For the olfactometric assays, each pair of a plant (orange jasmine or ‘Pera’ sweet orange) and control (clean air) in behavioral measurements (time spent in each odor field) was compared by using two-tailed, Wilcoxon matched-pairs signed rank test. All analyses were performed using the statistical software “R”, version 3.3.1^53^.

## Supplementary information

Supplementary files

## Data Availability

The datasets generated during and/or analyzed during the current study are available from the corresponding author on reasonable request.
