# Supplementary material for: Orange jasmine as a trap crop to control Diaphorina citri
Source: Sci Rep. 2019 Feb 14;9:2070. doi: 10.1038/s41598-019-38597-5 (PMC6376063; doi:10.1038/s41598-019-38597-5)
Supplement: Supplementary file 1 — Supplementary files [file 41598_2019_38597_MOESM1_ESM.docx]

**TITLE: Orange jasmine as a trap crop to control *Diaphorina citri***

**AUTHORS**

Arthur F Tomaseto^1^, Rodrigo N Marques^2^, Alberto Fereres^3^, Odimar Z Zanardi^1^, Haroldo XL Volpe^1^, Berta Alquézar^1,4^, Leandro Peña^1,4^ and Marcelo P Miranda^1*^

^1^Department of Entomology, Fund for Citrus Protection (FUNDECITRUS), 14807-040, Araraquara, São Paulo, Brazil.

^2^Centre of Nature Sciences, Federal University of São Carlos (UFSCAR), Buri, São Paulo, Brazil.

^3^Departamento de Protección Vegetal, Instituto de Ciencias Agrarias (ICA/CSIC), C/Serrano, 115 dpdo, 28006, Madrid, Spain.

^4^Instituto de Biología Molecular y Celular de Plantas (IBMCP), Consejo Superior de Investigaciones Científicas (CSIC), Universidad Politécnica de Valencia (UPV), 46022,

Valencia, Spain.

*Corresponding author: Marcelo Pedreira de Miranda, Fund for Citrus Protection (FUNDECITRUS), Av. Dr. Adhemar Pereira de Barros, 201, Vila Melhado, 14807-040, Araraquara, São Paulo, Brazil. Phone: +55 16 3301-7025. E-mail: [marcelo.miranda@fundecitrus.com.br](mailto:marcelo.miranda@fundecitrus.com.br)

|  |  |  | **Date 1** | | **Date 2** | |
| --- | --- | --- | --- | --- | --- | --- |
| **RT** | **Compound** | **CAS** | **Citrus** | **Orange jasmine** | **Citrus** | **Orange jasmine** |
| 18.55 | Ethyl benzene | 100414 | n.d. | 74285.22 ± 10859.47* | 92653.62 ± 92653.62 | 1141068.27 ± 663201.88* |
| 18.88 | p-xylene | 106423 | n.d. | 247807.86 ± 33034.57* | n.d. | 246767.91 ± 119680.38* |
| 20.57 | Ethyl senecionate | 638108 | n.d. | 1714644.74 ± 348114.19 | 34914.84 ± 34914.84 | 3873891.56 ± 2716812.89 |
| 21.05 | α-phellandrene | 99832 | 1934300.08 ± 1449445.30 | n.d.* | 3727249.89 ± 708105.12 | 110096.23 ± 92097.99* |
| 22.90 | β-phellandrene | 555102 | 9484869.48 ± 7920183.68 | 3997.53 ± 3997.53* | 5324996.03 ± 191732.61 | 110337.18 ± 90680.37 |
| 23.21 | Furan 2-pentyl | 3777693 | n.d. | 81054.92 ± 13519.36* | n.d. | 319671.57 ± 30890.69* |
| 23.30 | (-)-β-pinene | 18172673 | 1022169.14 ± 800292.14 | n.d.* | 1218274.70 ± 639913.82 | 15887.29 ± 15887.29* |
| 23.49 | Z-3-hexenyl acetate-1 | 3681718 | 302487.28 ± 168198.08 | 9920031.46 ± 1010688.94* | 3121436.13 ± 2933414.50 | 5597041.65 ± 1459437.26 |
| 23.57 | Z-3-hexenyl acetate-2 | 3681718 | n.d. | 120740.59 ± 21876.24* | n.d. | 189560.52 ± 28477.02* |
| 23.72 | Z-2-hexenyl acetate | 2497189 | n.d. | 156224.28 ± 12299.75* | n.d. | 125864.90 ± 13221.0* |
| 24.93 | d-limonene | 5989275 | 25683224.20 ± 20429507.81 | 446379.87 ± 15963.54* | 6382342.92 ± 2463135.55 | 555087.52 ± 101452.78 |
| 26.98 | Linalool | 78706 | 12309735.22 ± 9169615.82 | 319213.05 ± 43484.57* | 24439376.58 ± 7651514.09 | 1147619.88 ± 134121.64 |
| 27.03 | Unknown-1 |  | n.d. | 154117.57 ± 49642.17* | n.d. | 349243.48 ± 48177.39* |
| 27.11 | Unknown-2 |  | n.d. | 144542.04 ± 48013.94* | n.d. | 360033.85 ± 84775.70* |
| 29.59 | Butanoic acid 3-Z-hexenyl ester | 16491364 | n.d. | 80948.75 ± 23914.24* | 157702.12 ± 157702.12 | 431686.89 ± 46594.05* |
| 30.55 | Unknown-3 |  | n.d. | 151784.93 ± 38861.50* | n.d. | 409867.64 ± 59267.41* |
| 30.55 | α-terpineol | 98555 | 789487.64 ± 548762.17 | n.d.* | 1302187.18 ± 356582.43 | n.d.* |
| 31.07 | Unknown-4 |  | n.d. | 19652.72 ± 1422.61* | 244779.87 ± 244779.87 | 142421.88 ± 24457.00* |
| 31.20 | Unknown-5 |  | n.d. | 212091.95 ± 15380.47* | 53354.01 ± 53354.01 | 1615604.83 ± 77955.45* |
| 31.33 | Unknown-6 |  | n.d. | 28019.74 ± 3659.58* | n.d. | 132954.45 ± 21086.89* |
| 32.84 | Unknown-7 |  | n.d. | 112588.22 ± 39864.90* | n.d. | 184697.70 ± 18730.76* |
| 34.22 | Unknown-8 |  | n.d. | 43727.81 ± 4995.31* | n.d. | 28724.39 ± 17040.41* |
| 34.43 | Unk SQ-1 |  | n.d. | 70504.26 ± 12526.21* | n.d. | 31565.48 ± 15848.56* |
| 34.70 | Unk SQ-2 |  | n.d. | 138817.41 ± 24522.73* | n.d. | 30399.93 ± 5260.98* |
| 34.85 | γ-elemene | 339154915 | n.d. | 252144.01 ± 26103.82* | n.d. | 42277.06 ± 5816.19* |
| 35.22 | α-cubebene | 17699148 | 397692.64 ± 292425.92 | 20931820.72 ± 2718748.16* | 146838.88 ± 73231.08 | 3730002.90 ± 332956.02* |
| 35.73 | Unk SQ-3 |  | n.d. | 68657.51 ± 21558.66* | n.d. | 19129.19 ± 9923.43* |
| 36.07 | Germacrene D | 37839637 | n.d. | 265114.94 ± 24964.35* | n.d. | 60175.40 ± 4859.24* |
| 36.14 | Unknown-9 |  | n.d. | 191866.27 ± 29865.72* | n.d. | 64283.62 ± 2774.41* |
| 36.24 | α-copaene | 3856255 | 919589.23 ± 763682.37 | 17568574.93 ± 3028886.54* | 355487.38 ± 91741.61 | 3413414.87 ± 203731.03* |
| 36.47 | β-cubebene | 13744155 | n.d. | 11542247.31 ± 2155298.38* | n.d. | 820683.91 ± 109040.44* |
| 36.65 | Unk SQ-4 |  | n.d. | 215312.02 ± 13197.90* | n.d. | 26818.75 ± 8163.44* |
| 36.87 | Unk SQ-5 |  | n.d. | 54039.49 ± 54039.49* | n.d. | 17816.43 ± 8968.32* |
| 37.18 | Isocaryophyllene | 140072 | n.d. | 572266.47 ± 154274.83* | n.d. | 211808.04 ± 20479.46* |
| 37.25 | Unk SQ-6 |  | n.d. | 272472.53 ± 57919.15* | n.d. | 114813.48 ± 28426.12* |
| 37.38 | Unk SQ-7 |  | n.d. | 107605.51 ± 4777.68* | n.d. | 34507.31 ± 17649.97* |
| 37.51 | Unk SQ-8 |  | n.d. | 1007186.32 ± 169171.29* | n.d. | 174018.48 ± 73212.78 |
| 37.69 | β-caryophyllene | 87445 | 12368373.22 ± 9529886.07 | 95777420.84 ± 11500041.32* | 5313395.60 ± 1710557.90 | 20670905.05 ± 2221242.67* |
| 37.85 | Unk SQ-9 |  | n.d. | 1849536.76 ± 112260.66* | 158286.07 ± 158286.07 | 506788.93 ± 58983.96* |
| 37.93 | Unk SQ-10 |  | n.d. | 445356.21 ± 79911.62* | n.d. | 49896.87 ± 12947.51* |
| 38.05 | Unk SQ-11 |  | n.d. | 448588.50 ± 13987.49* | n.d. | 128737.24 ± 9765.81* |
| 38.17 | Unk SQ-12 |  | n.d. | 3147070.06 ± 133305.40* | n.d. | 1128665.72 ± 168240.25* |
| 38.83 | Unk SQ-13 |  | n.d. | 3255818.71 ± 340443.01* | n.d. | 858789.52 ± 72624.23* |
| 38.64 | α-humulene | 6753986 | 891502.55 ± 634475.67 | 21257950.60 ± 3708076.43* | 534992.81 ± 49055.51 | 4368695.82 ± 590103.11* |
| 38.81 | γ-muurolene | 6753986 | n.d. | 3482452.15 ± 299179.12* | n.d. | 1983955.24 ± 264105.12* |
| 38.97 | Zingiberene | 495603 | n.d. | 19420883.05 ± 3384778.72* | n.d. | 6501653.68 ± 563805.68* |
| 39.23 | Unk SQ-14 |  | n.d. | 6844459.17 ± 727778.38* | n.d. | 564122.84 ± 159575.11* |
| 39.34 | Unk SQ-15 |  | n.d. | 1415415.33 ± 31511.70* | 192022.55 ± 192022.55 | 520300.57 ± 106679.82* |
| 39.41 | Unk SQ-16 |  | n.d. | 1817877.77± 244385.79* | n.d. | 627128.82 ± 162552.86* |
| 39.52 | Unk SQ-17 |  | n.d. | 494257.48 ± 129002.06* | n.d. | 207964.10 ± 18546.32* |
| 39.60 | Unk SQ-18 |  | n.d. | 2397709.28 ± 302427.69* | n.d. | 408358.71 ± 89152.86* |
| 39.87 | β-cadinene | 523477 | n.d. | 18982299.91± 2778743.38* | 916106.77 ± 461292.07 | 6311840.77 ± 645413.85* |
| 40.01 | Unk SQ-19 |  | n.d. | 134634.21 ± 61327.51* | n.d. | 162083.04 ± 14740.77* |
| 40.11 | Unk SQ-20 |  | n.d. | 497698.28 ± 23038.25* | n.d. | 283955.53 ± 33456.89* |
| 40.36 | Unk SQ-21 |  | n.d. | 6204772.98 ± 723671.88* | n.d. | 2017469.37 ± 150489.33* |
| 40.44 | Unk SQ-22 |  | n.d. | 784499.45 ± 56172.76* | n.d. | 258142.91 ± 29241.62* |

**Supplementary Table-1 (Miranda)** Mean ± standard error of corrected area values (area per gram of fresh weight and hour) corresponding to volatile compounds emitted from *Murraya paniculata* and sweet orange (*Citrus ×aurantium* syn. *Citrus sinensis*) flushes. calculated from 3 samples at two sampling dates. For each date statistical analysis of differences between orange jasmine and orange was performed using LSD test and significance of differences (*P* < 0.05) are indicated by an asterisk. R.T. retention time; n.d. not detected; Unk-SQ. unknown sesquiterpene volatile compound.


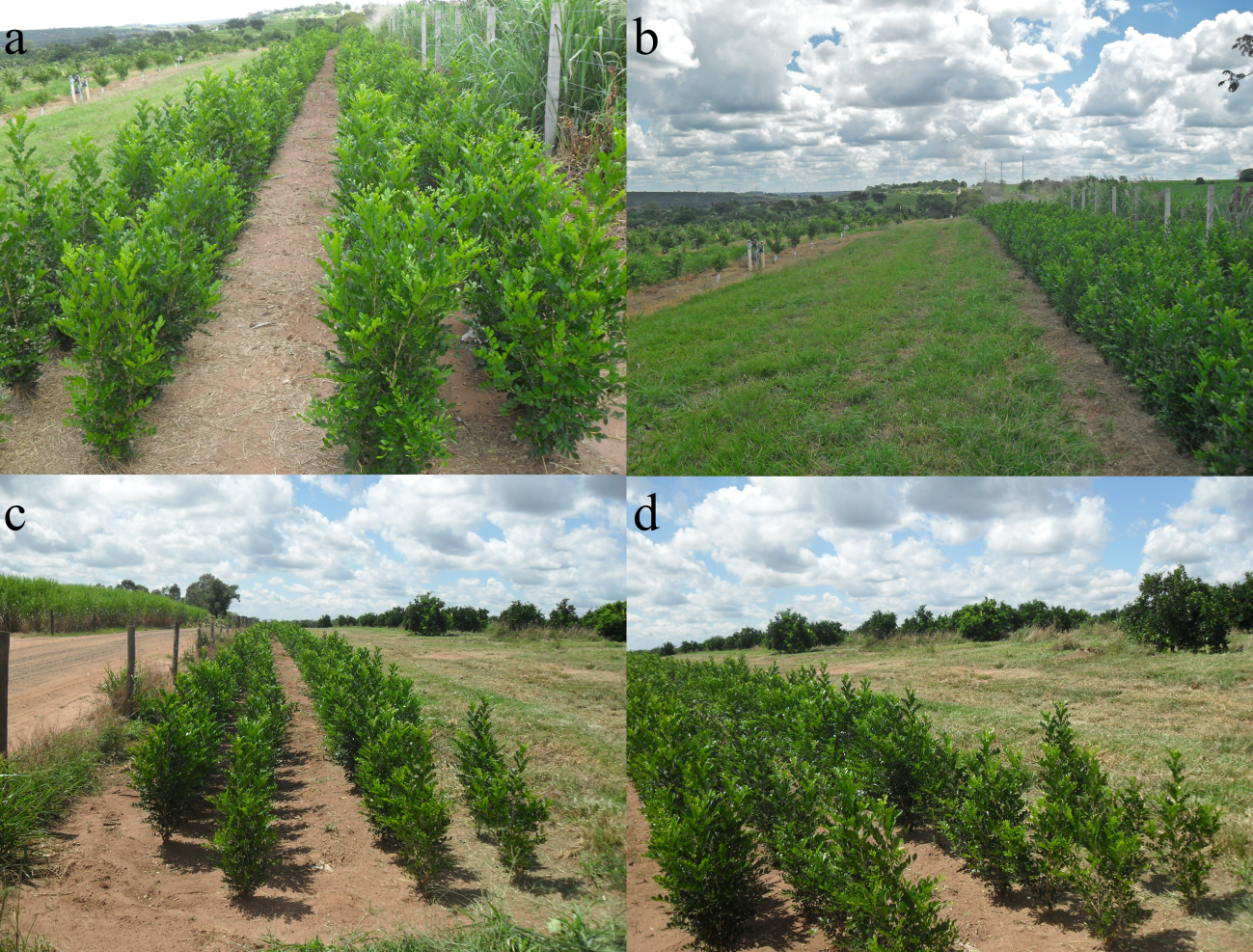


**Supplementary Figure-1 (Miranda)** Images of orange jasmine and citrus plants from the new (a and b) and stablished (c and d) orange orchards used to assess the effect of orange jasmine as trap crop on *D. citri* natural infestation and Huanglongbing incidence.

*
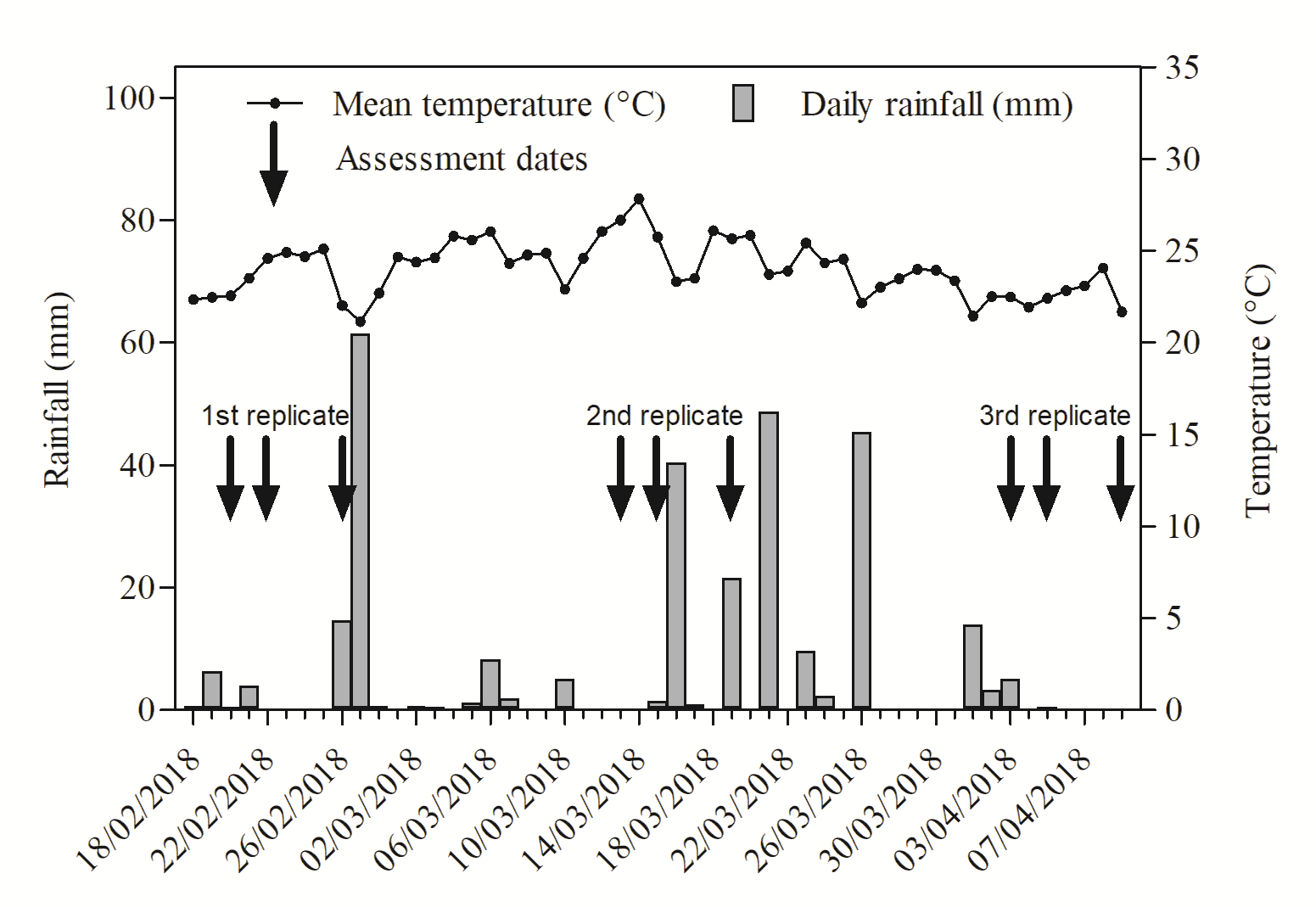
*

**Supplementary Figure-2 (Miranda)** Sum of daily rainfall (mm) and mean temperature (°C) along the evaluation of trap crop effect on *D. citri* settlement and dispersal. First, second and third black arrows in each replicate represent the assessment dates at 1, 3 and 7 days after insect release, respectively.
